# Effect of a Living–Learning Community on Nursing Student Outcomes—A Prospective Cohort Study

**DOI:** 10.3390/nursrep15050144

**Published:** 2025-04-28

**Authors:** Mary Bennett, Melissa Travelsted, Vickie Shoumake, Matthew Atkinson

**Affiliations:** School of Nursing and Allied Health, Western Kentucky University, Bowling Green, KY 42101, USA; melissa.travelsted@wku.edu (M.T.); vickie.shoumake@wku.edu (V.S.); matthew.atkinson@wku.edu (M.A.)

**Keywords:** BSN student success, living–learning community, student retention, workforce diversity

## Abstract

**Background/Objectives:** Prior studies have shown that most students seeking entry into a nursing program (also known as pre-nursing students) do not make it into the nursing profession, mostly due to failing one or more science courses in their first year of college. These students give up on nursing, dropping out of college or changing to a less challenging major. **Objectives:** We aimed to determine the effect of a living learning community (LLC) on the retention and success of students seeking entry into a Baccalaureate Science Nursing (BSN) program. **Methods:** The aim of this descriptive, prospective cohort project was to improve student retention and success by creating a living–learning community (LLC) for first-year students preparing to apply to a BSN program. The effectiveness of this intervention was determined by comparing retention and success for those in the LLC with those who were not in the LLC over a period of 4 years. **Results:** Fewer students in the LLC dropped out of or failed college (21% vs. 33%), fewer changed majors (24% vs. 27%), and more were ultimately admitted to the BSN program (42% vs. 36%) during their 3rd year of college. Of those not admitted within the study’s timeframe, there were more students still preparing to apply to a BSN program than those not in the LLC (13% vs. 3%). Regarding minority outcomes, fewer LLC underrepresented minority (URM) students dropped out of college or failed (29% vs. 43%), but more of them changed majors and remained in college, working towards a college degree in another field of study (43% vs. 29%). There was no apparent effect of participation in the LLC program on minority student nursing program admission success. An equal percentage (29%) of minority students from the LLC group and the non-LLC group were admitted to the BSN program during this study. **Conclusions:** The limitations affecting this study include the prohibition of large face-to-face gatherings during the initial part of this study and the lingering effects of the pandemic and infection control efforts on student learning outcomes. As reported in prior research, first-year nursing students have a high risk of failing or dropping out of college. However, the students who were able to participate in the LLC demonstrated better student outcomes than those who did not, resulting in more students who were able to move towards their goal of becoming a nurse.

## 1. Introduction

Universities across the country are facing declining enrollment and retention due to factors such as fewer high school graduates, underprepared applicants, limited student support resources, and high faculty workloads [1]. These issues are particularly acute in nursing education programs, which are subject to additional pressures related to nursing faculty shortages, difficulties regarding clinical placement, and the growing demand for nurses. In 2022, for the first time in 20 years, the number of US nursing applicants and students in BSN programs declined, and enrolment numbers in 2024 remained below pre-pandemic levels [2]. This underscores the urgent need for universities and nursing education programs to take measures to address these challenges and ensure there is a robust pipeline of qualified nurses for the future.

Previous research has shown that many students intending to apply to nursing programs leave college or change majors after failing one or more of the foundational science courses, often during their first year of college [3,4]. These losses are even more pronounced among underrepresented minority (URM) students, first-generation college students, and those requiring remedial coursework [5,6]. This emphasizes the need for early and effective interventions in order to improve first-year nursing student retention.

Living–learning communities (LLCs) have been recognized as a high-impact tool that supports first-year students’ success through enhanced engagement, improved academic performance, and a sense of belonging [1,4,7,8]. However, the results of studies on the effectiveness of LLCs regarding student retention are not uniformly positive. One study on several LLC cohorts reported mixed results, with one LLC in the study having a positive effect, while the other LLCs in the study did not improve student retention compared to that for other students at that university [9].

Another problem noted by prior LLC studies is that of student self-selection. Students who make the effort to join an LLC may be more motivated to succeed than those who do not. Therefore, the improved retention reported in some LLC studies could be related to the fact that LLC students were more motivated in the first place and not directly attributable to their being in an LLC [10].

While most studies support the effectiveness of LLCs in improving retention in the general student population, fewer studies have explored the impact of LLCs that are tailored specifically to nursing students or even students with health-related majors [7,8]. One pre-pandemic qualitative study of nursing students in an LLC highlighted the value these students placed on living with other nursing students and receiving mentorship from third- and fourth-year nursing students. The students in this study also valued having nursing students as their resident assistants who lived with them in their dorms. Some other themes that emerged from this study were mentoring and emotional support, mutual support, and being friends for life [7]. However, this prior study was not designed to look at more quantitative outcomes such as retention rates. Therefore, this study was developed to address the gap in the literature by examining the effect of a nursing-specific LLC on nursing student retention during the first year of college and the subsequent effect on BSN admission and other longer-term outcomes.

## 2. Materials and Methods

This project was reviewed for ethical concerns and received approval from the Western Kentucky University Institutional Review Board (IRB, #20-177). Demographic data and various student outcomes were collected using the university database (TOPNET), and plans were made to track student outcomes for up to 6 years from the time of admission to the university. This preliminary report presents the outcomes available as of May 2024.

The site of the current study was a large regional public university in the Southern United States, serving more than 15,000 students. Average graduation rate for the BSN program over 5 years was 85%, and average NCLEX pass rate over this same timeframe was 93%. Students at this university spend their first two years completing general education and nursing-specific science courses, including anatomy, chemistry, microbiology, and pathophysiology. These students are designated as pre-nursing majors, as they have declared nursing as their major but have not yet been admitted to the nursing program. Prior research conducted at this institution showed that roughly half of each pre-nursing cohort either dopped out of college, failed, or changed to a less demanding major within their first two years of college [6].

To address these issues, in this study, we implemented a nursing-specific LLC aimed at improving retention among first-year pre-nursing students. Due to limited space and resources, two LLC dormitory areas, which could hold about 24 students each, were reserved for the first year of this study. The effectiveness of the nursing-specific LLC was evaluated by comparing the 1st-year persistence, 3rd-year retention, admission to the BSN program, graduation rates, and National Council Licensure Examination (NCLEX) pass rates for those in the nursing LLC with those who were not in the nursing LLC. For this preliminary report, graduation rates and NCLEX pass rates are not yet available.

Due to the pandemic, face-to-face programing was suspended in March 2020, and all coursework was moved online. By August 2020, students were allowed to return to campus, and most classes resumed face-to-face meetings with social distancing in place. Large classes (more than 50 students) remained online. During the spring and summer of 2020, over 300 students who selected nursing as their planned major applied to the university. This group was informed about the development of a nursing-focused LLC and invited to apply for a reserved dormitory room within the nursing LLC.

Inclusion and exclusion criteria: Students included in this study had to have declared nursing as their intended major and applied to participate in the nursing-specific LLC. Students who applied to the LLC but changed their major prior to the first day of classes were excluded from this study. Dormitory rooms in two adjacent buildings were set aside for the nursing LLC participants. Because room availability was limited, LLC dormitory spaces were assigned on a first-come, first-served basis. Of the 83 students who applied to be in the nursing LLC, 13 changed majors prior to the first day of classes and were excluded from the study. The final sample included 70 pre-nursing students, with 37 placed in the LLC dormitory and 33 serving as the comparison group (not placed in the LLC but still enrolled as pre-nursing students).

To address concerns about LLC participants being more motivated and therefore possibly affecting study outcomes [10], students who applied to the nursing LLC but were not placed due to space limitations served as the comparison group. It was assumed that these students had similar motivation to those placed in the LLC and could thus offer a valid comparison if motivation alone were influencing study outcomes.

Interventions: As noted earlier, at the start of this study, pandemic restrictions on large, face-to-face classes and group gatherings were in place at the university. While students were allowed to stay in their dormitories, all classes with more than 50 students were held online, and most social gatherings were prohibited. Due to large section sizes, several of the required pre-nursing science courses were provided entirely online, with students attending the classes from their dormitory rooms or homes. For the smaller classes and labs, students were allowed to meet face to face, with masking required. These limitations on group meeting size also affected the activities that the LLC faculty had originally planned for the first part of this study.

Students in the pre-nursing LLC were assigned to a nursing advisor and housed in one of the two adjoining university residence halls. Common space available in the larger residence hall was used for various LLC events, including meetings with current nursing students, nursing faculty, and working nurses from the local hospitals. The local hospitals brought some incentives, such as hospital-branded items, and provided food for some of the meetings. However, fewer meetings than planned took place in the first 6 months of the study due to continued pandemic restrictions on large, face-to-face meetings.

One of the major interventions aimed at improving student outcomes for those in the nursing LLC was the use of linked courses, which meant that students in the LLC were advised to enroll in the same course sections during their first year. While not all students were enrolled in every linked course due to some having prior credits, all the students without prior credits were strongly encouraged to enroll in designated LLC course sections.

In the first semester, the core linked courses included the following:

- Chemistry for Health Professionals;
- College Algebra;
- Developmental Psychology.

In the second semester, the core linked courses were as follows:

- Anatomy and Physiology I;
- Introduction to Nursing.

The goal was to create a sense of community by advising LLC students to register for the same liked course sections. This allowed for the creation of a type of built-in study group, as most of the students in the LLC studied for the same exams at the same time, particularly in the core linked courses. In addition, tutoring and mentoring were provided, particularly for the high-failure-rate courses such as anatomy and physiology. During the first semester, tutoring was provided weekly by a graduate teaching assistant (GTA) who was majoring in biology. For the second semester, a nursing student tutor was added for support for the Introduction to Nursing course as well as additional support for anatomy and physiology. Students in the LLC also met with junior and senior nursing students and the nursing LLC faculty supervisor for mentoring at various events throughout the year, facilitating additional engagement, connection, and encouragement.

Comparison Group: To determine if the nursing LLC and the various forms of support implemented improved outcomes, we compared the nursing LLC student (*n* = 37) outcomes with the outcomes for students in the comparison group (*n* = 33), all university freshmen (*n* = 2886), all incoming pre-nursing students (*n* = 292), and all students who participated in one of the other university LLCs (*n* = 229).

To ensure comparability between the students in the nursing LLC and the 33 students in the comparison group, a thorough examination of both groups was conducted. In addition to all students in both groups being in pre-nursing and expressing interest in joining the nursing LLC, demographic information, high school GPA, high school rating, college entrance exam (ACT) scores, first-generation status, and Pell grant eligibility were compared between the two groups. Pell grant eligibility indicates lower family income. It is worth noting that neither group contained students who spoke English as a second language, so this factor was not a variable in this study.

To assess the quality of the high schools attended by students in both groups, high school ratings were obtained from Great Schools, a website that rates schools on a scale of 1–10, with 10 being the highest rating possible (https://www.greatschools.org, accessed on 8 August 2023). This organization compares schools based on standardized college entrance exam scores (ACT and SAT), state-required test scores, and disparities between high- and low-income students and majority and minority students’ outcomes. Graduation rates, the number of students enrolled in advanced placement courses, college admission rates, and retention rates of students within their first two years of college were also taken into consideration. It is important to note that these school ratings were not available for the five students who attended private schools and the one student who was homeschooled.

## 3. Results

Sample Comparison: The two groups of students (the LLC and comparison groups) had very similar demographics, with the same number of underrepresented minorities and first-generation students in each group (see Table 1). There were more out-of-state students and Pell-grant-eligible students in the LLC group. The non-LLC comparison group had slightly higher high school GPAs and college entrance exam (ACT) scores. However, a *t*-test of the interval level data available for these students did not show any statistically significant differences (*p* < 0.05) between these two groups of subjects prior to the start of the study.

As this is a descriptive study examining the effect of LLC participation on commonly measured program outcome data such as persistence and retention rates, the results of this study are presented here using percentages. In this study, persistence denotes the number of students who started in August of 2020 and returned for enrollment the next semester (January 2021). Retention numbers include those students who started college in August of 2020 and were still enrolled as of May 2024.

Persistence and retention data from both the larger university group consisting of all incoming first-year students for Fall 2020 (*n* = 2886) and all first-year students who were in one of the university LLCs during the Fall of 2020 (*n* = 229) were included to provide a big-picture overview of what was happening at the university during this study. This report also includes comparison data for all pre-nursing students who started during the Fall of 2020 (*n* = 292), plus the subset of pre-nursing students who were in the nursing LLC (*n* = 37) and the comparison group (*n* = 33).

As demonstrated in the demographics Section, the 37 LLC students and the 33 who were interested but did not join the LLC had similar demographics and test scores. Given that students who are interested in joining an LLC for whatever reason might be more motivated than the overall group of incoming first-year students or incoming pre-nursing students, the comparison group provided another way to determine if the LLC was making a real difference or if the improvement in the retention data was due to participant self-selection bias [10].

**Figure 1:**
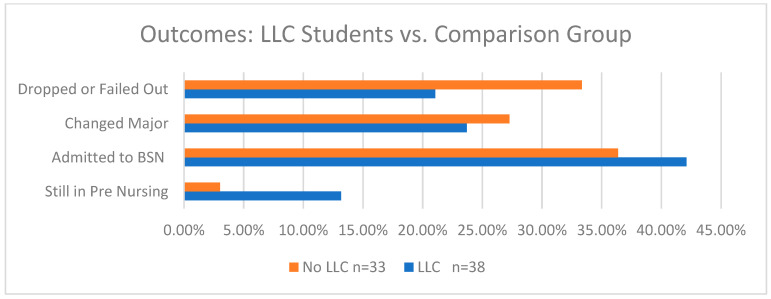
Outcomes for the living–learning community (LLC) vs. those for the comparison group.

Figure 1 shows that compared to the comparison group data, the students in the nursing LLC during their first year of college had fewer drop/fail outcomes (21% vs. 33%), slightly fewer changed to a major other than nursing, (24% vs. 27%), and more were admitted to the BSN program (42% vs. 36%). More of these students were also still preparing to apply to the BSN program in comparison to those who were not in the LLC (13% vs. 3%).

**Figure 2:**
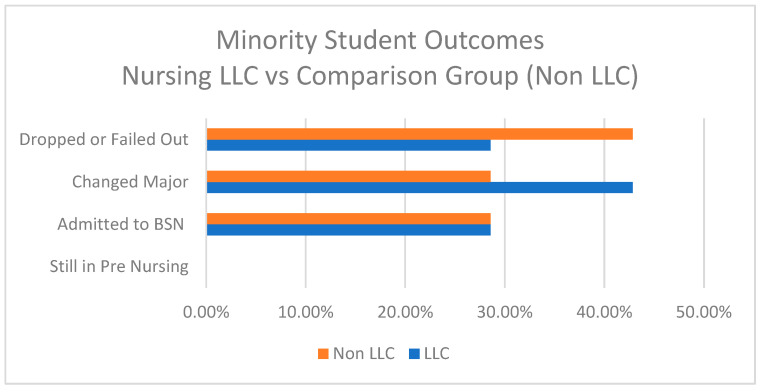
URM outcomes for the living-learning community (LLC) vs. URM in the comparison group.

As demonstrated in Figure 2, a direct comparison of the underrepresented minority students reveals that fewer of the minority students in the LLC dropped out or failed, (29% vs. 43%), but more of them changed to a different major (43% vs. 29%). There was no difference in the percentages for those who were ultimately admitted to the BSN program (29% for both groups). By the end of the Spring 2024 semester, none of the minority students in either group remained in pre-nursing; they had either been admitted to the nursing program, changed to another major, or dropped out of the university. While this result is disappointing in terms of the LLC leading to more minority students entering the BSN program, it demonstrates that there was better overall college retention for the minority students in the LLC compared to those not in the LLC.

**Figure 3:**
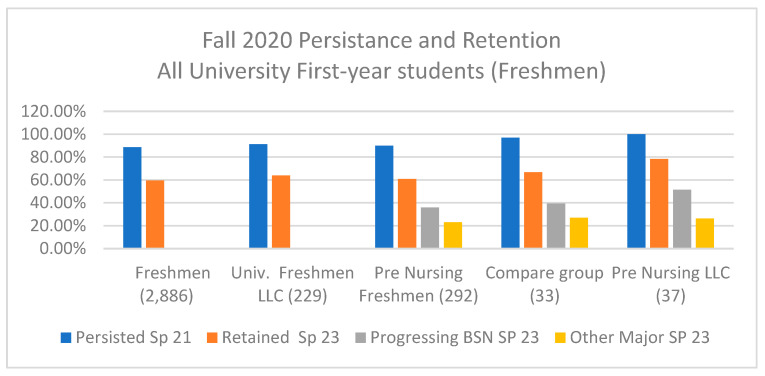
Comparison of the intervention and comparison groups with all incoming first-year students (freshman), all university LLC students, and all pre-nursing students.

When comparing the results for the LLC nursing students with those concerning the larger incoming university group, it is evident that students participating in any of the university LLCs groups consistently demonstrated higher persistence and retention rates compared to those who did not take part in an LLC (see Figure 3). The findings also reveal that pre-nursing students not in the LLC generally achieved academic results similar to the general university student outcomes. However, it is interesting to note that the comparison group for this study, which consisted of students who expressed interest in joining the nursing LLC but were not placed in an LLC dormitory, still outperformed the overall group of pre-nursing students. This observation suggests that students who express interest in joining an LLC may possess qualities that make them better students overall, supporting the idea that there may be some self-selection bias influencing the comparison of LLC student outcomes with those of the general student population [10]. Nevertheless, when comparing the outcomes of the nursing LLC with those of the comparison group, improvements in persistence, retention, and admission to the nursing program were still evident.

## 4. Discussion and Limitations

This project has several limitations that should be considered. Firstly, it is important to acknowledge that this study was conducted at a single site, which may limit the generalizability of the findings to other institutions or populations. Replicating this study at multiple sites would improve the robustness of the results. Additionally, the project was started during the COVID-19 pandemic, which introduced various restrictions and challenges. The prohibition of indoor or large, face-to-face gatherings; difficulties in hiring tutors; and the prohibition of face-to-face course meetings affected the initial start of the study and some of the interventions. These circumstances may have influenced student outcomes and should be considered when interpreting the results. Also, this report focuses on student outcomes for the pre-nursing curriculum and initial progress in the BSN program only. An ongoing study will include additional student outcomes such as graduation rates from the BSN program and NCLEX pass rates.

An additional limitation pertains to student participation in the various LLC activities, including the tutoring sessions. Despite offering tutoring for the more difficult courses, many students did not participate in the tutoring sessions, particularly those offered by the non-nursing-graduate teaching assistants. While nearly 85% of the LLC students attended at least one tutoring session, by the end of the first semester, attendance had dropped to less than 30% of students in attendance. Attendance was higher at sessions offered by junior- and senior-level nursing students, as the LLC students felt that they had more in common with the nursing students than with the graduate-level science students.

Despite these limitations, the outcomes thus far align with prior research indicating that pre-nursing students have a high risk of failure or dropping out, particularly with respect to their first-year science courses. The results from this study also indicate that the students who participated in the pre-nursing LLC had better outcomes compared to those who did not participate. This pattern holds true when considering first-generation and underrepresented minority students, although the sample size was small, and the results for admission to the BSN program did not improve for URM students. URM minority students in the LLC were less likely to drop out of college or fail but more likely to switch to a less challenging major.

Importantly, it should be emphasized that being part of the nursing LLC did not influence admission selection for the nursing program. The program uses a formula based on GPA, science GPA, and the anatomy and physiology subscale of a nursing entrance exam (HESI A2) to rank students. The development of this objective selection process and formula has been described in a prior paper [3]. Another factor in the admissions process was the decrease in student applications to the nursing program during the study timeframe. It is possible that students preparing to apply to the nursing program were influenced by the pandemic and an increased level of awareness of the challenges of the nursing profession. As a result of these challenges, fewer qualified applicants were ready to enter the nursing program during the study timeframe. Due to the decreased number of qualified applicants from January 2022 through January 2023, all qualified applicants were admitted into the BSN program during this timeframe, resulting in 100% acceptance rates during the first part of this study.

## 5. Conclusions

This study found that participation in a nursing-specific living–learning community (LLC) was associated with improved retention among first-year pre-nursing students. LLC participants were less likely to drop out of college, fail foundational science courses, or change majors, increasing their chances of being admitted to the BSN program. These findings support the notion that a nursing-specific LLC can be an effective strategy for supporting early retention and goal attainment among pre-nursing students.

However, the impact of the LLC appeared to be different for the underrepresented minority (URM) students. While the URM participants in the LLC were less likely to fail or leave college during their freshman year, they were more likely to change their majors. As a result, there was no difference in the BSN program admission rates between the URM students who participated in the LLC and the URM students in the comparison group. This suggests that while LLCs may mitigate some barriers, additional support may be needed to address challenges that disproportionally affect URM students.

In response to these findings, the nursing LLC program has been continued and expanded, with space for up to 75 students being reserved annually from 2021 to 2025. Funding from a state grant has allowed us to add additional dorm rooms, plus more student engagement activities and more student peer tutors. Due to LLC student feedback, we have stopped employing non-nursing graduate teaching assistants as tutors. We are now using junior and senior nursing students for the LLC tutoring sessions, and we have added a nursing student tutor for Pathophysiology, as this course also has a high failure rate. Ongoing evaluation of key outcomes—including first-year student retention, pass rates for nursing pathophysiology, admission to the BSN program, graduation rates, and NCLEX pass rates—will be conducted in a longer-term study to further assess and document the effectiveness of a nursing-specific living–learning community model with respect to these important outcomes.

## Figures and Tables

**Table 1:** LLC vs. comparison-group demographics.

Demographic Variable	LLC Group	Comparison Group
Number of Participants	37	33
Underrepresented Minority	7 (18.92%)	7 (21.21%)
Males	3 (8.10%)	2 (6.06%)
Out-of-State Students	15 (40.54%)	10 (30.30%)
First Generation	9 (24.32%)	9 (27.27%)
Pell-Grant-Eligible	15 (40.54%)	11 (33.33%)
Mean High School Rating	5.44	5.77
Mean High School GPA	3.53	3.60
Mean ACT	20.89	21.84
Mean Science ACT	20.76	22.03

## Data Availability

The raw data supporting the conclusions of this article will be made available by the authors on request. These data are part of an ongoing study and currently have student ID codes to allow for continued follow-ups. The university will not allow the release of data containing these codes, so prior to release, these data will need to be de-identified.

## Public Involvement Statement

There was no public involvement in any aspect of this research.

## Guidelines and Standards Statement

This manuscript was drafted in consideration of the SQUIRE 2.0 guidelines for quality improvement research and using the template provided by the journal “Nursing Reports”.
